# Supplementary material for: Genome-wide analysis of the role of the antibiotic biosynthesis regulator AbsA2 in Streptomyces coelicolor A3(2)
Source: PLoS One. 2019 Apr 10;14(4):e0200673. doi: 10.1371/journal.pone.0200673 (PMC6457490; doi:10.1371/journal.pone.0200673)
Supplement: S1 Fig — The green sequences represent restriction enzyme cut sites incorporated into the termini for ease of cloning. The black sequence represents the absA1- absA2 intergenic region, the red sequence represents the DNA which encodes the triple-flag tag and the blue sequence represents the absA2 coding sequence. (PDF) [file pone.0200673.s001.pdf]

ATCGGATCCCACCAGGGAAGGATCGG ATG GAC TAC AAG GAC CAC GAC GGC GAC TAC AAG
GAC CAC GAC ATC GAC TAC AAG GAC GAC GAC GAC AAG ATG ATT CGC GTA CTG CTC
GCC GAC GAC GAG ACC ATC ATC AGG GCC GGG GTT CGC TCC ATC CTG ACG ACC GAA
CCG GGC ATC GAG GTG GTC GCC GAG GCG TCC GAC GGG CGG GAG GCG GTG GAA CTG
GCC CGC AAG CAC CGG CCC GAC GTG GCC CTG CTC GAC ATC CGG ATG CCG GAG ATG
GAC GGC CTG ACG GCC GCG GGT GAG ATG CGG ACC ACC AAC CCG GAC ACC GCG GTC
GTC GTC CTC ACC ACC TTC GGG GAG GAC CGG TAC ATC GAA CGG GCC CTG GAC CAG
GGC GTG GCC GGG TTC CTG CTC AAG GCG TCC GAT CCG CGG GAC CTG ATC TCC GGC
GTA CGG GCC GTG GCG TCC GGC GGC TCC TGC CTC TCC CCG CTG GTG GCG CGG CGG
CTG ATG ACC GAG CTG CGC CGG GCC CCC TCA CCG CGC TCG GAG GTG TCG GGG GAG
CGC ACG ACG CTG CTG ACC AAG CGG GAG CAG GAG GTC CTC GGC ATG CTG GGG GCC
GGG CTG TCG AAC GCG GAG ATC GCG CAG CGG CTG CAC CTG GTC GAG GGC ACG ATC
AAG ACG TAT GTC AGC GCC ATC TTC ACC CAG TTG GAG GTC CGC AAC CGG GTG CAG
GCG GCG ATC ATC GCG TAC GAG GCG GGA CTG GTG AAG GAC GCC GAC CTC AAC CGT
TAG TCTAGAGAT

Sequence of the N-terminally triple flag tagged *absA2* and upstream region.
The green sequences represent restriction enzyme cut sites incorporated into the termini for ease of cloning. The black sequences represent the *absA1-absA2* intergenic region, the red sequence represents the DNA which encodes the triple-flag tag and the blue sequence represents the *absA2* coding sequence.
