# Supplementary figures and images for: Genome-wide analysis of the role of the antibiotic biosynthesis regulator AbsA2 in Streptomyces coelicolor A3(2)
Source: PLoS One. 2019 Apr 10;14(4):e0200673. doi: 10.1371/journal.pone.0200673 (PMC6457490; doi:10.1371/journal.pone.0200673)

Fig.\_S2. PCR-based verification of *absA2* deletion

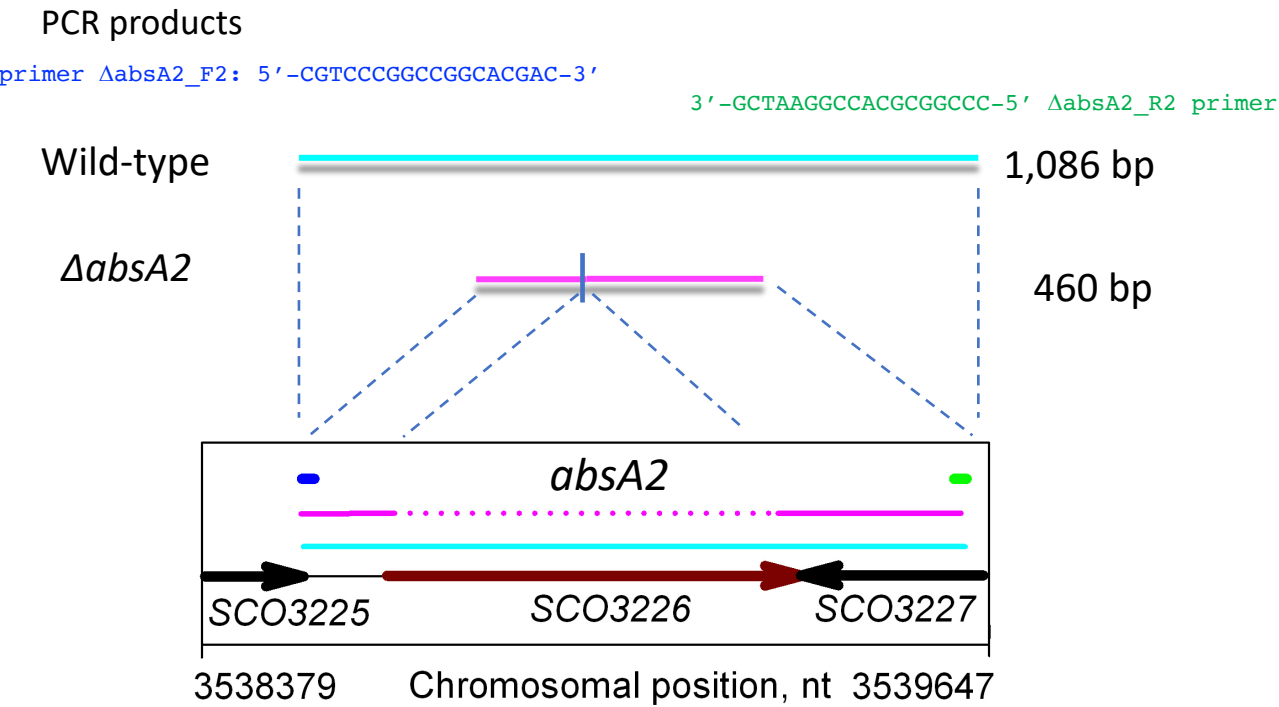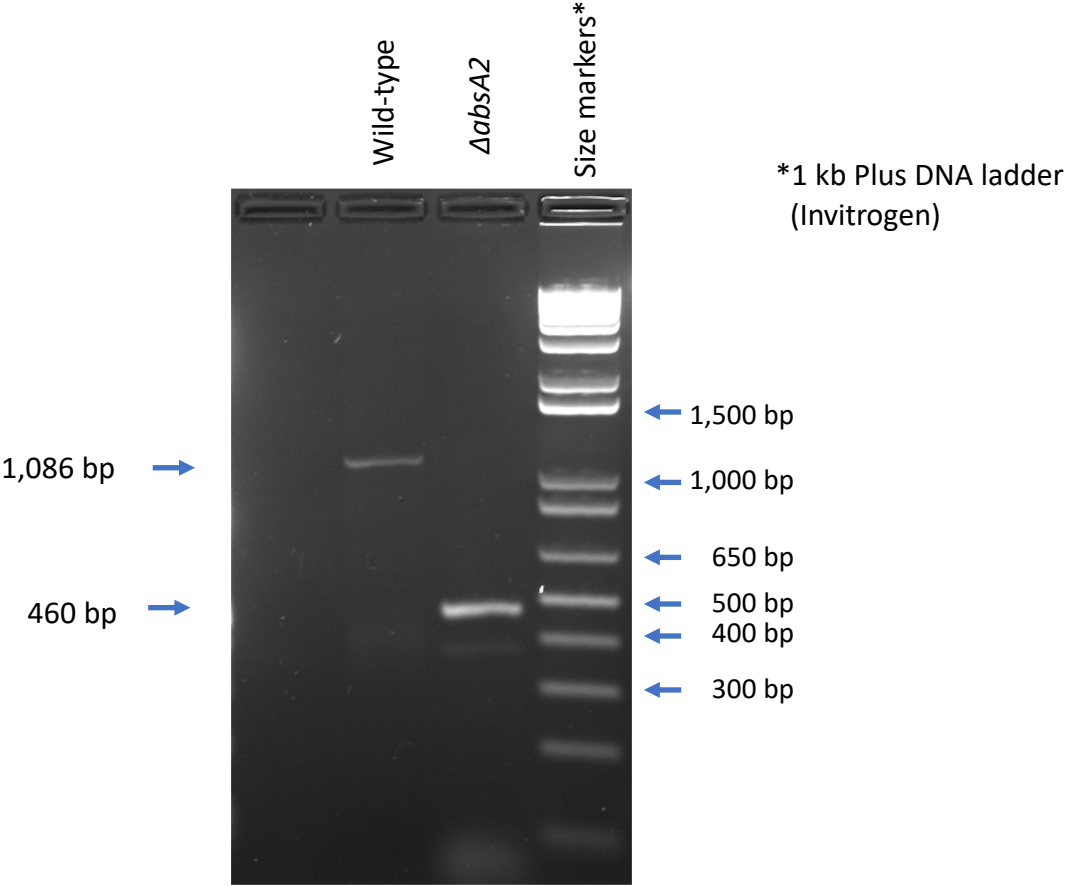

Supplement: S2 Fig — Upper panel: Schematic diagram illustrating the absA genomic region and the location of the absA2 deletion. The locations of the PCR primer target sequences and sizes of the MT1110 wild-type PCR product (1,086 bp) and the ΔabsA2 PCR product (460 bp) are shown. Lower panel: Agarose gel photograph illustrating PCR products of the expected sizes generated from MT1110 wild-type and the ΔabsA2 strain. (PDF) [file pone.0200673.s002.pdf]

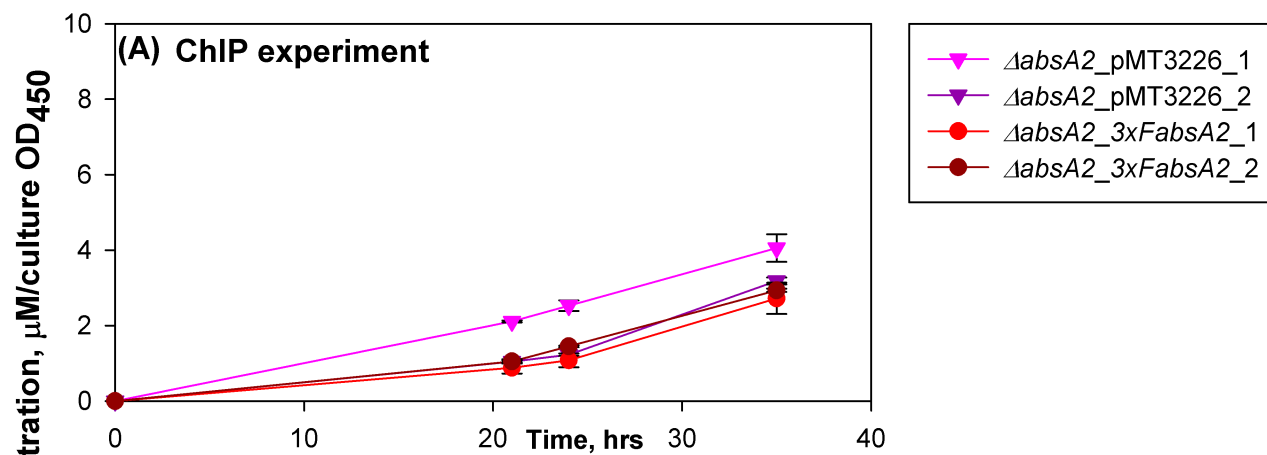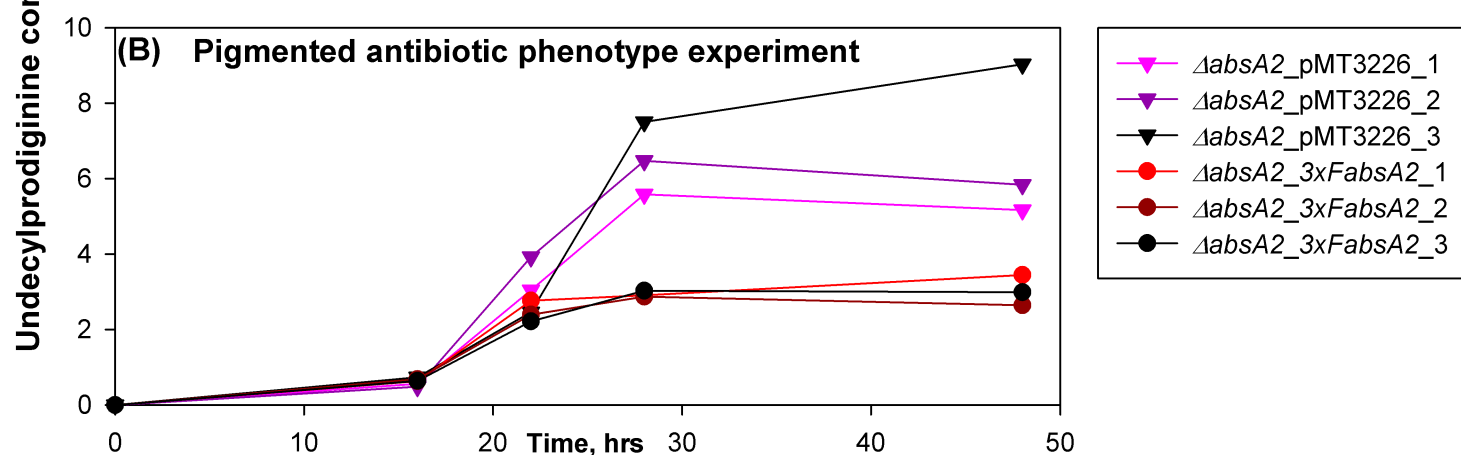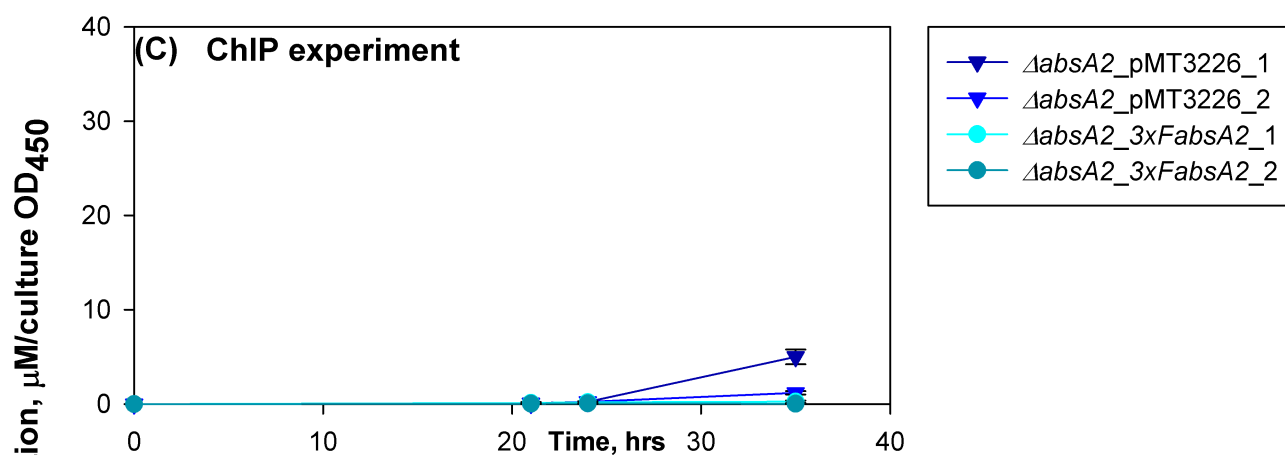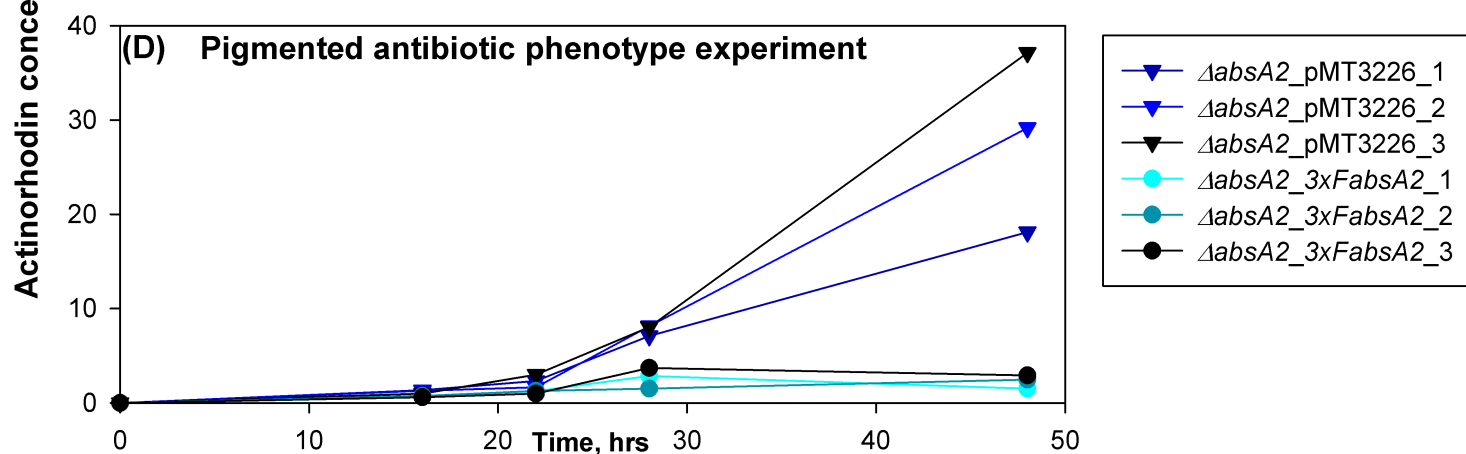

Supplement: S3 Fig — Actinorhodin (ACT) and undecylprodiginine (RED) assays from replicate cultures of strains of MT1110 ΔabsA2 (pMT3226) and MT1110 ΔabsA2 (pMT3226::3xFabsA2) used in the main chIP/transcriptomic experiment: panels (A) & (C) and from the independent small-scale pigmented antibiotic production experiment: panels (B) & (D). (PDF) [file pone.0200673.s003.pdf]
