# Supplementary material for: Genome-wide analysis of the role of the antibiotic biosynthesis regulator AbsA2 in Streptomyces coelicolor A3(2)
Source: PLoS One. 2019 Apr 10;14(4):e0200673. doi: 10.1371/journal.pone.0200673 (PMC6457490; doi:10.1371/journal.pone.0200673)
Supplement: S2 Table — List of genes which are significantly differentially expressed between MT1110 ΔabsA2 (pMT3226) and MT1110 ΔabsA2 (pMT3226::3xFabsA2) at the 14 h, 18 h and 35 h time-points (Rank Product pfp <0.15). Genes in red are repressed in MT1110 ΔabsA2 (pMT3226::3xFabsA2) relative to MT1110 ΔabsA2 (pMT3226). Genes highlighted in green are activated in MT1110 ΔabsA2 (pMT3226::3xFabsA2) relative to MT1110 ΔabsA2 (pMT3226). Genes which possess clusters of ChIP-enriched microarray probes in MT1110 ΔabsA2 (pMT3226::3xFabsA2) relative to MT1110 ΔabsA2 (pMT3226) are highlighted by green/red background shading; the method used for clustering and scoring these clusters or ‘regions of interest’ is detailed in Materials and Methods. Genes of the act biosynthetic gene cluster are highlighted by red borders and genes of the cda biosynthetic gene cluster are highlighted by green borders. (PDF) [file pone.0200673.s008.pdf]

14hrs		18hrs		35hrs	
SCO0892	SCO6932	SCO0131	SCO0209	SCO0409	SCO0955
SCO1089		SCO0268	SCO0678	SCO0736	SCO3425
SCO1454		SCO0396	SCO0955	SCO0892	SCO3427
SCO1963		SCO0560	SCO1598	SCO0919	SCO3428
SCO3216		SCO0892	SCO4174	SCO1048	SCO5071
SCO3223		SCO0919	SCO4652	SCO1195	SCO5072
SCO3224		SCO0920	SCO4653	SCO1541	SCO5073
SCO3226		SCO0921	SCO5680	SCO1795	SCO5074
SCO3764		SCO1048	SCO6624	SCO1811	SCO5075
SCO3765		SCO1195	SCO7040	SCO1908	SCO5078
SCO4214		SCO1196		SCO1963	SCO5079
SCO4562		SCO1122		SCO2014	SCO5080
SCO4739		SCO1123		SCO2113	SCO5081
SCO4813		SCO1290		SCO2148	SCO5085
SCO5632		SCO1565		SCO2198	SCO5086
SCO6056		SCO1648		SCO2205	SCO5087
SCO7470		SCO1908		SCO3083	SCO5088
		SCO1963		SCO3108	SCO5089
		SCO1968		SCO3226	SCO5090
		SCO1969		SCO3357	SCO5092
		SCO2014		SCO3661	SCO5650
		SCO2286		SCO3668	
		SCO2348		SCO3670	
		SCO2878		SCO3671	
		SCO2995		SCO4187	
		SCO2996		SCO4189	
		SCO3210		SCO4198	
		SCO3211		SCO4200	
		SCO3215		SCO4214	
		SCO3217		SCO4471	
		SCO3220		SCO4540	
		SCO3221		SCO5013	
		SCO3222		SCO5014	
		SCO3226		SCO5028	
		SCO3227		SCO5140	
		SCO3229		SCO5366	
		SCO3235		SCO5414	
		SCO3236		SCO5583	
		SCO3239		SCO5584	
		SCO3242		SCO5632	
		SCO3243		SCO6054	
		SCO3244		SCO6480	
		SCO3246		SCO6808	
		SCO3247		SCO7481	
		SCO3248		SCO7658	
		SCO3249			
		SCO3546			
		SCO3790			
		SCO4140			
		SCO4141			
		SCO4142			
		SCO4152			
		SCO4212			
		SCO4227			
		SCO4228			
		SCO4229			
		SCO4230			
		SCO4442			
		SCO4540			
		SCO4612			
		SCO4881			

		SCO4908			
		SCO5013			
		SCO5140			
		SCO5366			
		SCO5367			
		SCO5632			
		SCO5746			
		SCO6053			
		SCO6054			
		SCO6313			
		SCO6691			
		SCO7481			
		SCO7631			
		SCO7697			
		SCO7717			
